# Environmental pollution and deaths due to stroke in a city with low levels of air pollution: ecological time series study

**DOI:** 10.1590/1516-3180-2014-1326733

**Published:** 2014-09-02

**Authors:** Camila Trolez Amancio, Luiz Fernando Nascimento

**Affiliations:** I Medical Student. Department of Medicine, Universidade de Taubaté (Unitau), Taubaté, São Paulo, Brazil; I PhD. Assistant Professor, Department of Medicine, Universidade de Taubaté (Unitau), Taubaté, São Paulo, Brazil

**Keywords:** Stroke, Air pollutants, Particulate matter, Mortality, Sulfur dioxide, Acidente vascular cerebral, Poluentes do ar, Material particulado, Mortalidade, Dióxido de enxofre

## Abstract

**CONTEXT AND OBJECTIVE:**

Little has been discussed about the increased risk of stroke after exposure to air pollutants, particularly in Brazil. The mechanisms through which air pollution can influence occurrences of vascular events such as stroke are still poorly understood. The aim of this study was to estimate the association between exposure to some air pollutants and risk of death due to stroke.

**DESIGN AND SETTING:**

Ecological time series study with data from São José dos Campos, Brazil.

**METHODS:**

Data on deaths due to stroke among individuals of all ages living in São José dos Campos and on particulate matter, sulfur dioxide and ozone were used. Statistical analysis was performed using a generalized additive model of Poisson regression with the Statistica software, in unipollutant and multipollutant models. The percentage increase in the risk of increased interquartile difference was calculated.

**RESULTS:**

There were 1,032 deaths due to stroke, ranging from 0 to 5 per day. The statistical significance of the exposure to particulate matter was ascertained in the unipollutant model and the importance of particulate matter and sulfur dioxide, in the multipollutant model. The increases in risk were 10% and 7%, for particulate matter and sulfur dioxide, respectively.

**CONCLUSION:**

It was possible to identify exposure to air pollutants as a risk factor for death due to stroke, even in a city with low levels of air pollution.

## Abstract (pt)

**CONTEXTO E OBJETIVO:**

Pouco se tem discutido sobre o aumento do risco de acidente vascular cerebral após exposição a poluentes do ar, principalmente no Brasil. Os mecanismos pelos quais a poluição pode influenciar a ocorrência de eventos vasculares, tais como acidente vascular cerebral, ainda são pouco compreendidos. O objetivo deste estudo foi estimar a associação entre exposição a alguns poluentes do ar e risco de morte por acidente vascular cerebral.

**TIPO DE ESTUDO E LOCAL:**

Estudo ecológico de séries temporais com dados de São José dos Campos, Brasil.

**MÉTODOS:**

Dados sobre mortes por acidente vascular cerebral em indivíduos de todas as idades que vivem em São José dos Campos e sobre material particulado, dióxido de enxofre e ozônio foram utilizados. A análise estatística utilizou modelo aditivo generalizado de regressão de Poisson com o *software* Statistica, em modelos "*unipollutant*" e "*multipollutant*". Foi calculado o percentual de aumento no risco para o aumento da diferença interquartil.

**RESULTADOS:**

Houve 1.032 mortes por acidente vascular cerebral, que variou de 0 a 5 por dia. A significância estatística da exposição ao material particulado em modelo "unipoluente" e a importância do material particulado e dióxido de enxofre no modelo "multipoluente" foram verificadas. O aumento do risco foi de 10% e 7%, respectivamente para material particulado e dióxido de enxofre.

**CONCLUSÃO:**

Foi possível identificar exposição a poluentes do ar como um fator de risco para morte por acidente vascular cerebral, mesmo em uma cidade com baixos níveis de poluição do ar.

## INTRODUCTION

The deleterious effects of exposure to environmental pollution have been studied recently in Brazil, focusing mainly on diseases of the respiratory and circulatory systems and the increased risk of morbidity and mortality was confirmed.^1^^-^ 4 However, little has been discussed about the increased risk of stroke, particularly in Brazil. An association between stroke and exposure to air pollutants has been shown, in Europe, Asia and Brazil, mainly covering hospitalization due to this cause.^5^^-^ 8 Recent studies have also shown that the risk of stroke is also greater in regions with low levels of pollutants.^6^^,^ 9

Stroke deserves special attention because it is the leading cause of disability worldwide, compromising the quality of life of those who fall ill as a consequence of it. Thus, stroke, characterized by rapid loss of neurological function due to ischemia or hemorrhaging of brain vessels, is a serious public health problem.^10^ In 2009, in Brazil, about US$ 200 million were invested in clinical treatments for affected patients.^11^

The pollutants studied and most commonly associated with deleterious effects on human health are particulate matter (PM_10_), sulfur dioxide (SO_2_) and ozone (O_3_). PM_10 _is a mixture of solid and liquid particles suspended in air; SO_2_ is generated from combustion of fossil elements and O_3_ is formed by reaction between ultraviolet radiation, nitrogen oxides and hydrocarbons emitted by vehicles.^12^

However, the mechanisms through which air pollution may influence occurrences of vascular events such as stroke remain poorly understood. It has been shown in relation to cardiovascular mortality that fine particulate matter acts through mechanisms that include systemic inflammation, accelerated atherosclerosis and altered cardiac autonomic function.^13^

## OBJECTIVE

Due to the uncertainties that still exist on this subject, the aim of the present study was to estimate the association between exposure to major air pollutants and risk of death due to stroke in a medium-sized city in Brazil, with low levels of air pollution.

## METHODS

This was an ecological time series study covering a five-year period (January 1, 2005, to December 31, 2009), using information on deaths due to stroke among individuals of all ages living in the city of São José dos Campos. Diagnoses were coded in accordance with the 10th International Classification of Diseases (ICD-10), taking into account the definitions I-60 to I-69. This information was obtained from the Mortality Information System (SIM).

São José dos Campos is located between São Paulo and Rio de Janeiro, at latitude 23º 11' south and longitude 45º 53' west, 600 meters above sea level. It is an important industrial and commercial center in this region and has approximately 600,000 inhabitants. It has about 1100 industrial establishments with emphasis on automobile manufacturing, aerospace and pharmaceutical industries, and an oil refinery (information available on www.ibge.gov.br). The city is crossed by the Dutra highway, which is the most important highway in Brazil, with heavy traffic involving about 130,000 vehicles per day, including cars, trucks and buses. 

The pollutants included in the analysis were particulate matter with an aerodynamic diameter < 10 μm (PM_10_), sulfur dioxide (SO_2_) and ozone (O_3_). The data were obtained from the São Paulo State Environmental Agency (CETESB), which has a measuring station in downtown São José dos Campos. Meteorological data such as relative humidity, average temperature and atmospheric pressure were obtained from the Foundation for Science, Technology and Space Applications (FUNCATE). The pollutant concentration values used were taken from a CETESB report.^14^

We built distributed lag models covering the times of 0 to 5 days after exposure, because the acute effects of exposure to air pollutants can manifest several days after this exposure.

The analysis was adjusted for average temperature, humidity and atmospheric pressure. The pollutant data were analyzed in continuous unipollutant and multipollutant models, such that all three pollutants were firstly studied separately, and subsequently together.

The statistical analysis used was the generalized additive model (GAM) of Poisson regression, with the Statistica software. The analysis yielded relative risks (RR) and 95% confidence intervals (CI) for each lag structure constructed.

In the multipollutant model, we calculated the percentage increase in risk caused by increases in the interquartile difference (IQD) in pollutant concentration, by means of the formula:

PI = [exp (coeff VIQPOL *) - 1] * 100

where PI is the percentage increase in the risk of death due to stroke and VIQPOL is the difference between the third and first quartiles of the pollutant concentration. A significance level of 5% was used for all the analyses. 

## RESULTS

During the study period, there were 1032 recorded deaths due to stroke among individuals of all ages living in São José dos Campos, thus generating a daily mean of 0.56 (SD = 0.77) and a range from 0 to 5.

In relation to air pollutants, the mean PM_10_ was 24 mg/m^3^ (SD = 12.4), without exceeding the established standard of an acceptable annual mean of 50 μg/m^3^. The annual daily mean for SO_2_ was 3.5 μg/m^3^ (SD = 2.6), without exceeding the established value of 80 μg/m^3^. The maximum daily O_3_ concentration for one hour was 209 μg/m^3^, which exceeded the maximum acceptable for this length of time per day, which is 80 μg/m^3^. The interquartile differences obtained were 15 μg/m^3^ for PM_10_, 2 μg/m^3^ for SO_2_ and 41 μg/m^3^ for O_3_. These data are summarized in Table 1.

**Table 1:** Descriptive analysis on the variables under assessment. São José dos Campos, Brazil, 2005-2009

	Mean	SD	Minimum	Maximum	IQD
Deaths	0.56	0.77	0	5	
PM_10 _(mg/m^3^)	23.96	12.41	6	100	15
SO_2 _(mg/m^3^)	3.45	2.57	0.5	27	2
O_3 _(mg/m^3^)	80.62	32.24	5	209	41
Humidity (%)	83.52	7.25	54.0	100	
Temperature (ºC)	20.7	2.45	13.7	27.9	
Pressure (mbar)	948.2	3.33	938	960	

SD = standard deviation

IQD = interquartile difference.

**Figure 1:**
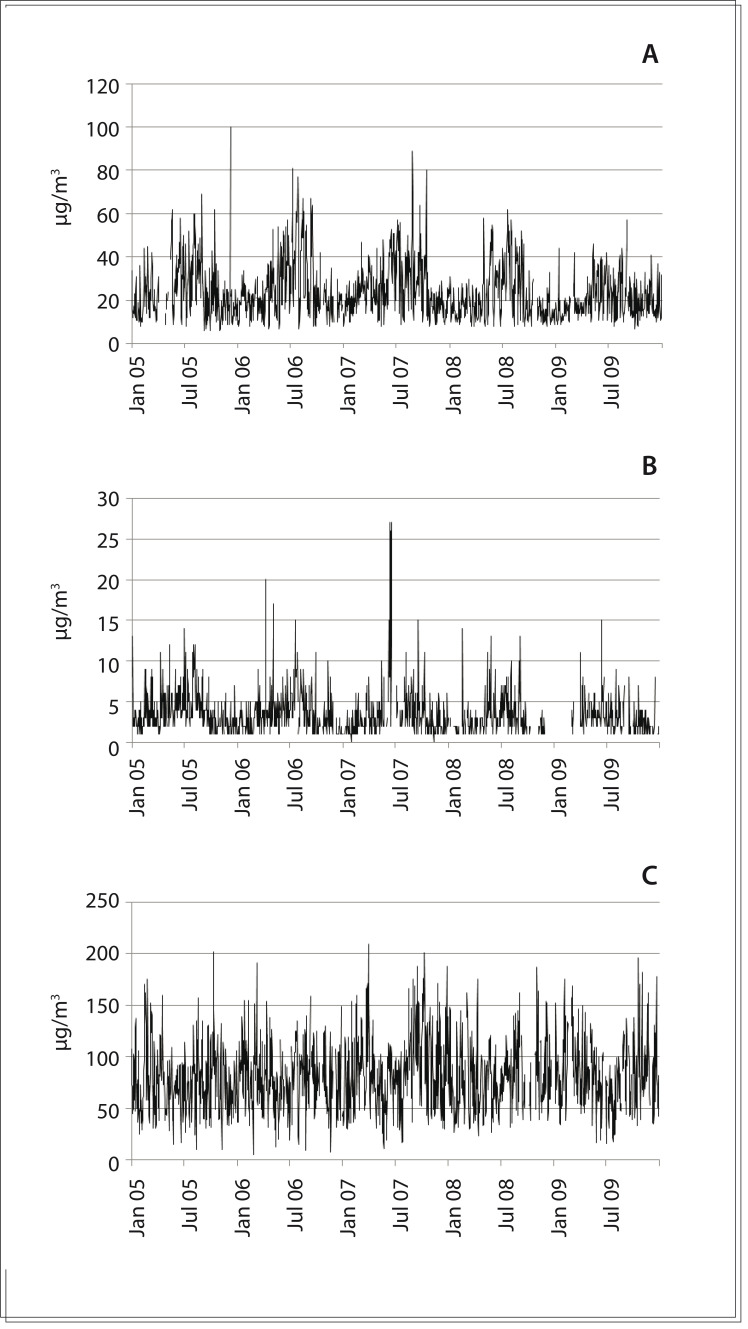
Pollutant levels in μg/m3: (A) particulate matter; (B) sulfur dioxide; (C) ozone. São José dos Campos, Brazil, 2005-2009.

Figure 1 shows the variation in pollutant levels over the five years of the study. It is interesting to note that there was seasonal variation of the pollutants particulate matter and sulfur dioxide, which increased in level during the colder periods of the year.

Regarding data gaps, there were data on the pollutant levels for particulate matter on 90 days (4.9%), sulfur dioxide on 332 days (18.1%) and ozone on 136 days (7.4%). However, these data gaps did not damage the final outcome from the study.

A statistically significant association between exposure to particulate matter and death due to stroke (RR: 1.005; 95% CI: 1.000 to 1.011) on the same day as the exposure (lag 0) was noted in the unipollutant model. No significant association was found for the other pollutants.

**Figure 2:**
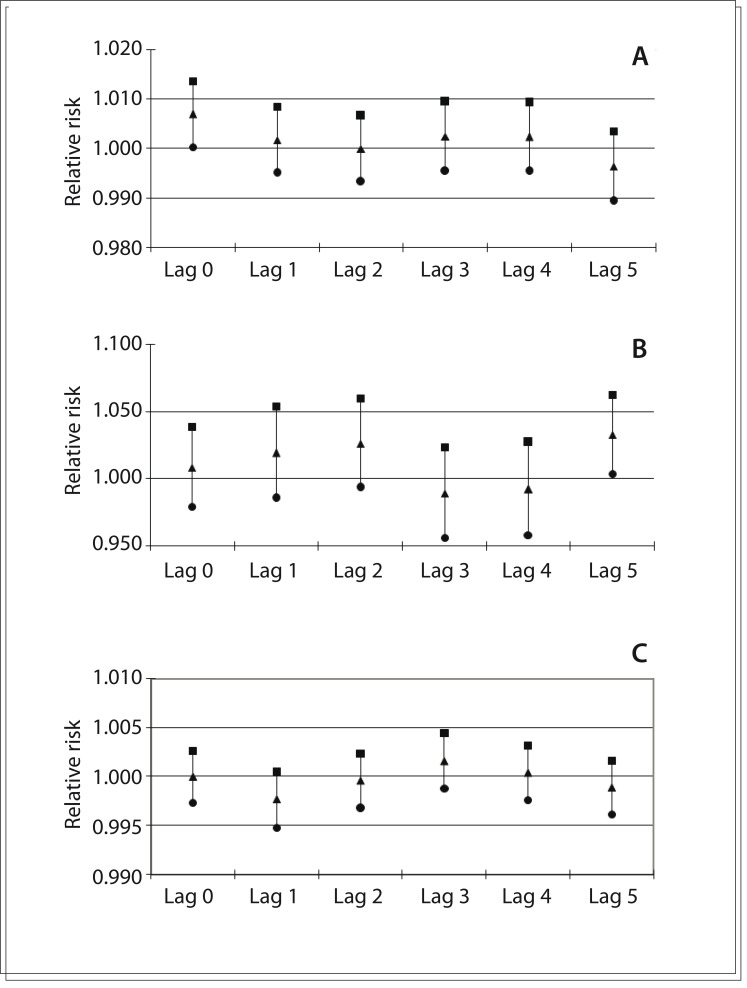
Relative risks and 95% confidence intervals in relation to multipollutant model; (A) particulate matter; (B) sulfur dioxide; (C) ozone. São José dos Campos, Brazil, 2005-2009.

Figure 2 shows the relative risks for each lag structure and their respective 95% confidence intervals for death due to stroke in the multipollutant model. Deaths due to stroke were significantly associated with exposure to particulate matter on the same day as the exposure (RR: 1.007; 95% CI: 1.000 to 1.014); and with exposure to sulfur dioxide on the fifth day after exposure (lag 5) (RR: 1.033; 95% CI: 1.004 to 1.063).

**Figure 3:**
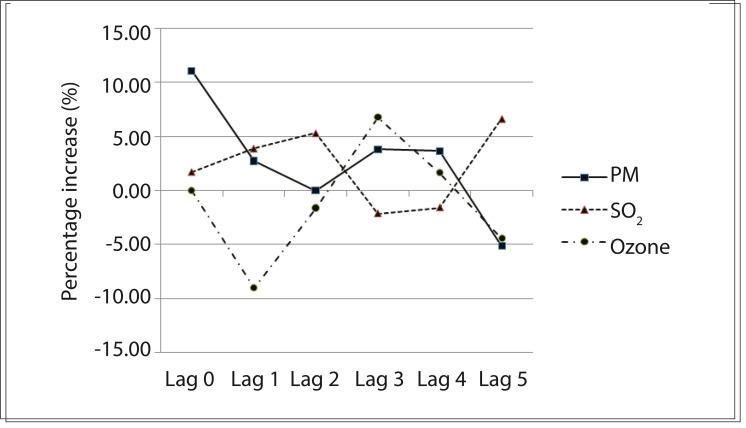
Percentage increase in relative risk, in multipollutant model according to lags. São José dos Campos, Brazil, 2005-2009.

In relation to increases in the interquartile difference for the pollutant in the multipollutant model, we observed that there was a statistically significant increased risk of death of approximately 10% on the same day as the exposure to PM_10_ and 7% on the fifth day after exposure to SO_2_, as shown in Figure 3.

## DISCUSSION

An increased risk of hospitalization due to stroke was observed in the city of São José dos Campos. This is one of the first cities in Brazil for which estimates for the risk of death from this cause have been made.8 This finding deserves attention because little research has been conducted worldwide on the association between exposure to air pollutants and stroke.

The use of lags to estimate the risk of death is widely used in methodologies worldwide, while the number of lags analyzed has varied.^6^^,^ 15 We chose to use five days of lag because of the possibility that this might produce a more comprehensive approach, thereby agreeing with the Korean methodology.^16^

The statistical analysis for this approach was the generalized additive model of Poisson regression, which had already been established for this type of analysis, since the variables under assessment are counted variables. However, some studies have addressed this issue by making use of a generalized linear model.^4^^,^ 8 It has already been found that both the additive and the linear model show consistent results.^17^

To select cases of death due to stroke obtained from the Mortality Information System (SIM), we used the International Classification of Diseases in its tenth revision (ICD-10), including diagnoses classified as I60 to I69 together for analyses. The deaths due to stroke were not separated into ischemic or hemorrhagic types, which has been recommended in some studies,^7^^,^ 15 because most of the deaths recorded during the period covered by this study were categorized as unspecified stroke. 

This study showed that significant exposure to particulate matter was a risk factor for death due to stroke both in the unipollutant and in the multipollutant model. There was a 10% higher risk of a greater interquartile difference, which was more than what was found by Wellenius et al. (1.03%) for ischemic stroke.^15^ A positive association between stroke mortality and the level of exposure to fine particulate matter (PM_2.5_) on the same day and preceding day, and also to ultrafine particles on the preceding day, was reported in a study developed in Helsinki, during the summer season.^6^

The activation of microglia in response to air pollution and identification of particulate matter both in brain capillaries and in parenchyma suggest that particulate matter is capable of interacting with cells and crossing the blood-brain barrier.^18^^,^ 19 Recent advances have shown that systemic inflammation has a significant impact on brain tissue. It has been accepted that air pollution causes proinflammatory signals originating in peripheral organs such as the lungs, which transfer inflammation to the brain.^20^ As an example, one study showed that in response to tumor necrosis factor alpha (TNF alpha) during inflammation of a peripheral organ, animals appeared to recruit larger quantities of circulating monocytes to the brain.^21^

In the present, sulfur dioxide was significant in the multipollutant model only in relation to the five-day lag, unlike the findings of another study, in which this pollutant was a significant risk factor for exposure on the same day, for inclusion in the unipollutant model (RR: 1029; 95% CI: 1.000-1.060).^8^ This might be explained by the different focus of that study, since hospital admissions and not deaths were taken into consideration. In Seoul, Korea, there was a 2.9% higher risk of a greater interquartile difference, two days after exposure in the unipollutant model, with a mean SO_2 _concentration of 30 μg/m^3^, i.e. well above what was found in the present study.^16^ Although it was expected that the increased risk of mortality would be greater in the area where the pollutant concentration was highest, this was not observed in the present study, which showed a percentage increase of 7%. This might be explained by the difference in methodology, such that one used a unipollutant model and the other used adjustment by other pollutants. In another study, which used a generalized linear model, sulfur dioxide was found to be a statistically significant risk factor for death due to stroke in the fifth and sixth moving means.^4^ This result is more similar to what was found in the present study, thus showing the importance of exposure to sulfur dioxide on the fifth day after exposure when using both the moving mean and the lag methodology.

In turn, ozone was not a statistically significant risk factor in the present study, and this coincides with research using a generalized linear model for hospital admissions.^8^ The 5% higher interquartile difference in the third lag found in this study was close to what was found by Hong et al. (2.9% on the same day as the exposure), but without statistical significance.^16^

It is important to remember that the present study only addressed stroke events that resulted in death. Another point to note is that the database used is susceptible to human error. On the other hand, this database (Datasus), which contains secondary records, is greatly used in studies on air pollution and its health effects. Ecological studies like the present study may have limitations in terms of confounders and ecological bias; moreover, epidemiological studies do not identify causes. 

There may be delays between symptoms and death, or misdiagnosis or error in codings used for deaths, since the diagnosis of this disease is clinical and there may not have been any autopsy or examination like tomography to provide confirmation. Moreover, we considered homogeneous concentrations of pollutants, rather than individual exposure. The present study did not include or analyze any interactions with risk factors inherent to human health that have already been established for stroke. It is important to emphasize that these results do not determine causality, but rather, they indicate a possible association between exposure to pollutants and deaths due to stroke. 

The strength of this study lies in the fact that it demonstrates the effect of air pollutant exposure on human health, even at low concentrations. Another positive point to be highlighted is that studies in medium-sized cities in which air pollutant concentrations are measured can use this methodology to assess these effects and to establish public health policies.

## CONCLUSION

This study showed that there was an association between exposure to major air pollutants and deaths due to stroke, and it serves as an important tool for public health management.
